# Social anxiety increases visible anxiety signs during social encounters but does not impair performance

**DOI:** 10.1186/s40359-019-0300-5

**Published:** 2019-04-23

**Authors:** Trevor Thompson, Nejra Van Zalk, Christopher Marshall, Melanie Sargeant, Brendon Stubbs

**Affiliations:** 1 Department of Psychology, School of Health and Social Care, University of Greenwich, London, SE9 2UG UK; 2 Faculty of Engineering, Dyson School of Design Engineering, Imperial College, London, SW7 2AZ UK; 3 Start2Stop Addictions Treatment Centre, London, SW7 3HG UK; 4 University of Greenwich, London, SE9 2UG UK; 5 Institute of Psychiatry, Psychology and Neuroscience, King’s College London, De Crespigny Park, London, SE5 8AF UK

**Keywords:** Social anxiety, Social performance, Social discomfort, Sex differences

## Abstract

**Background:**

Preliminary evidence suggests that impairment of social performance in socially anxious individuals may be specific to selective aspects of performance and be more pronounced in females. This evidence is based primarily on contrasting results from studies using all-male or all-female samples or that differ in type of social behaviour assessed. However, methodological differences (e.g. statistical power, participant population) across these studies means it is difficult to determine whether behavioural or gender-specific effects are genuine or artefactual. The current study examined whether the link between social anxiety and social behaviour was dependent upon gender and the behavioural dimension assessed within the same study under methodologically homogenous conditions.

**Methods:**

Ninety-three university students (45 males, 48 females) with a mean age of 25.6 years and varying in their level of social anxiety underwent an interaction and a speech task. The speech task involved giving a brief impromptu presentation in front of a small group of three people, while the interaction task involved “getting to know” an opposite-sex confederate. Independent raters assessed social performance on 5 key dimensions from Fydrich’s Social Performance Rating Scale.

**Results:**

Regression analysis revealed a significant moderate association of social anxiety with behavioral discomfort (e.g., fidgeting, trembling) for interaction and speech tasks, but no association with other performance dimensions (e.g., verbal fluency, quality of verbal expression). No sex differences were found.

**Conclusions:**

These results suggest that the impairing effects of social anxiety within the non-clinical range may exacerbate overt behavioral agitation during high demand social challenges but have little impact on other observable aspects of performance quality.

## Background

Social anxiety disorder (SAD) is a common psychiatric disorder, with up to 1 in 8 people suffering from SAD at some point in their life [1]. SAD is linked to reduced quality of life, occupational underachievement and poor psychological well-being, and is highly comorbid with other disorders [2]. Mounting evidence suggests that social anxiety exists on a severity continuum [3], and that social anxiety that is not severe enough to warrant a diagnosis of SAD may still produce significant individual burden [4].

There is little evidence to suggest that social anxiety may negatively affect others’ perceptions of agreeableness or warmth [5]. However, if social anxiety impairs an individual’s ability to function effectively in common performance situations such as job interviews, presentations and other social challenges [6], this could cause or maintain feelings of failure and inadequacy and even affect career success [7]. Cognitive models [8] predict that social anxiety could impair social competence by increasing self-focused attention and consuming attentional resources necessary for effective communication. On the other hand, social anxiety can also lead to a willingness to engage in socially-facilitative behavior such as polite smiling, head nodding and avoiding interruption, which can facilitate interaction and lead to more favorable impression of another’s social behavior [9].

While socially anxious individuals reliably *believe* their social behavior is deficient, the existence of actual impairment has been the subject of a fair amount of debate [10]. Empirical studies that have examined the association between social anxiety and behavior in response to social challenge tasks in both clinical and non-clinical samples have produced inconsistent findings. Strahan and Conger [11], for example, compared the responses of 26 men with low social anxiety with 27 men reporting clinical levels of social anxiety on the Social Phobia and Anxiety Inventory in their response to a simulated job interview. Observer ratings of videotaped interviews indicated no group differences in overall social competence ratings. Rapee and Lim [12] found that, when asked to give a brief impromptu speech, a group of 28 individuals with SAD did not differ in observer ratings of overall performance relative to a group of 33 non-clinical controls. Similar null results have been reported in a non-clinical sample of males on overall impressions of social skill on an opposite-sex “getting to know you” task [13], and in a sample of 110 schoolchildren participating in a two-minute impromptu speech where observers rated video recordings for global impressions and “micro-behaviors” (e.g., clarity of speech, ‘looking at the camera’) [14].

However, a number of other studies have identified a link between social anxiety and impaired social behavior. Levitan et al. [15] found that patients with SAD were rated significantly more poorly on observer ratings of voice intonation and fluency during a three-minute speech compared to controls. Other studies have also found patients with SAD to be rated more poorly by observers on adequacy of eye contact and speech clarity [16] and as exhibiting more “negative social behaviors” (e.g. awkwardness) during conversations [17, 18]. In a non-clinical study of 48 women, Thompson and Rapee [18] found individuals with high social anxiety to be rated more poorly during an opposite-sex “getting to know you” task on summed measures of molecular (e.g. voice quality, conversational skill) behaviors and on overall impression.

A recent review by Schneider and Turk [10] suggests that the apparently variable link between social anxiety and behavior is likely to be influenced by differences across studies in factors such as statistical power, sample characteristics and the type of behavioral assessments used. Assessment measures, for example, have ranged from global impression ratings to composite scores of molecular behaviors (e.g., smiling frequency, eye contact), and it may be that social anxiety impairs certain social behaviors but not others. There is some evidence that social anxiety may selectively exacerbate observable anxiety signs but have little impact on performance ‘quality’ (e.g. factors central to effective communication) [14, 19]. Schneider and Turk [10] note, however, that it is difficult to identify a coherent pattern that identifies which aspects of performance may be impaired by social anxiety and which may not and this is additionally complicated by differences in study designs. Furthermore, where associations of social anxiety across multiple behavioral dimensions have been examined within the same study, where they are evaluated under the same conditions, these differences have rarely been compared statistically which limits the reliability of the current evidence for selective deficits in social behavior [20].

Norton [21] also notes that studies using exclusively female samples have often found stronger associations of social anxiety with behavioral deficits than studies with male samples, consistent with the argument that gender-role expectations may lead to more deleterious effects of social anxiety in women [22]. Again, however, it is impossible to determine with any certainty whether more pronounced effects of social anxiety in studies with females is attributable to moderating effects of gender or some other difference in study characteristics. Unfortunately, few studies have directly compared males and females, or different performance dimensions, within the same study where there is greater methodological homogeneity.

This study aimed to assess social behavior during social challenges in a non-clinical sample of individuals varying in their levels of social anxiety. We used speech and interaction tasks, as these represent different types of commonly-encountered social challenges. Performance was assessed by independent raters using Fydrich’s Social Performance Rating Scale, which consists of five separate dimensions of social competence. The aim of the study was to examine whether social anxiety is associated with impaired social behavior, and in particular: (1) whether impairment occurs only for specific dimensions of behavior, and (2) whether impairing effects are greater in females.

## Method

### Participants

The sample consisted of 93 participants (45 males and 48 females) with a mean age of 25.6 years (*SD* = 7.7, Range = 18–53). Males (*M* = 26.5 years) and females (*M* = 24.7 years) did not differ significantly with respect to age, *t (86)* = 1.12, *p* = .26. Scores on the Social Phobia Scale were lower for males (M = 17.1, SD = 9.68) compared to females (M = 22.7, SD = 12.7), and this difference reached statistical significance, *t* (91) = 2.36, *p* = .02.

The mean SPS score of the current sample was 20.0 (*SD* = 11.6, range = 2–48). Compared to McNeil et al.’s (1995) reference data, this is significantly lower than the mean SPS score of individuals with SAD, *M* = 32.8, *SD* = 14.8, *t* (57) = 5.86, *p* < .001, but significantly higher than undergraduates, *M* = 13.4, *SD* = 9.6, *t* (144) = 3.69, *p* < .001, and community volunteers, *M* = 12.5, *SD* = 11.5, *t* (141) = 3.70, *p* < .001. The mean age of these comparison groups was higher (SAD sample *M* = 36.5 years, community sample *M* = 33.2 years, with age data not reported for undergraduates) than the current sample.

An exclusion criterion of previous acquaintance with the experimenters was implemented, as familiarity may have reduced the effectiveness of the social challenge tasks as anxiety inductions. A recruitment request was e-mailed to all students at Greenwich University which stated that “volunteers are sought to take part in a paid (£10) study which will involve filling in some questionnaires, engaging in a conversation task and talking to others about a set topic, giving your views”.

### Anxiety and social behavior scales

Mattick and Clarke’s *Social Phobia Scale* (SPS)^1^ was used to assess level of trait social anxiety. The SPS consists of 20 items rated on a five-point (0–4) scale, with higher scores indicating greater social anxiety. The scale has been shown to reliably assess social anxiety in both non-clinical and clinical populations [23]. The SPS has previously demonstrated good test-retest reliability, internal consistency and convergent validity [24, 25] and exhibited high internal consistency (Cronbach’s α = .89) for the current data.

*State anxiety* was assessed in order to verify that the speech and interaction tasks resulted in increased anxiety relative to participants’ baseline anxiety. Baseline anxiety was assessed with a single self-report item that asked respondents to indicate their current anxiety on a scale of 1–10. State anxiety was also assessed immediately prior to the commencement of each task (participants had been provided with task details a few minutes earlier), and immediately after each task where participants were asked to rate the anxiety they had felt during the task itself. Single-item assessments of state anxiety have shown good reliability and convergent validity [26].

The *Social Performance Rating Scale* (SPRS) [27] was used to rate the participant on the following five dimensions: Gaze - adequacy of eye contact, Vocal Quality – warmth, clarity and enthusiasm demonstrated in verbal expression, Length – low level of monosyllabic speech/excessive talking, Discomfort – low levels of behavioral anxiety (e.g., fidgeting, trembling, postural tension), and Flow - verbal fluency (including the ability to incorporate information provided by the conversation partner smoothly into the interaction). The flow item was not used in the assessment of the speech task, as the rating descriptors for this component are specific to conversation. All SPRS items were rated on a 5-point scale and scored so that higher scores represented more effective social performance. Detailed descriptive anchors accompany each rating point to facilitate scoring; for example, Vocal Quality, “5 (Very Good) = Participant is warm and enthusiastic in verbal expression without sounding condescending or gushy”. The SPRS has shown excellent inter-rater reliability, internal consistency, convergent, discriminant and criterion validity [27, 28]. Agreement across the three raters assessing the speech task was examined with an intraclass correlation (ICC). An absolute-agreement model was used [29], which is a stringent test requiring both high inter-rater correlations and minimal discrepancy in actual rating values to produce a high ICC. Analysis revealed ICC’s = .64–.86 for individual SPRS dimensions (all p’s < .001), suggesting good rater agreement [30]. Scores were therefore averaged across raters for each individual SPRS dimension for the speech task. Similar means (range: 3.4–3.8) and standard deviations (range: 0.7–1.1) were observed across SPRS components for both interaction and speech tasks.

### Speech task

Participants were given 3 min to prepare a speech presenting a persuasive argument on their choice of one of the following topics: “sometimes it is ok to lie, discuss” or “can any crime be justified?”. Participants were told they would be presenting in front of a small audience and that they should try to keep going for 3 min although they could terminate the task at any point. Three confederates (one male and two female) comprised the “audience” for the speech task, with the same three-confederate audience used for each participant. The confederate audience had previously undertaken a number of trial sessions with several undergraduate volunteers acting as participants where they had practiced maintaining neutral facial expressions.

### Interaction task

Participants were told that they would shortly be introduced to someone and that they would have 3 min to find out as much as they could about this person, although they could terminate the task at any time. The conversation partner was an experimental confederate, who was of the opposite-sex in order to maximize socially-evaluative challenge [6]. The same male confederate was used for each female participant, and the same female confederate was used for each male participant, with the one male and one female confederate taken from the pool of three confederates used in the speech task. Confederates had previously undertaken a number of trial sessions amongst each other and with undergraduate volunteers, where they practiced giving minimal responses, avoiding asking questions and maintaining neutral facial expressions [6]. Nobody other than the participant and the confederate was present during the interaction task when the experiment began.

### Procedure

To put participants in a relaxed state for a reliable assessment of baseline state anxiety, and to provide time for the experimenter to prepare the social challenge tasks, participants watched a 5-min relaxation video showing images of various seascapes accompanied by relaxing sounds. They then immediately completed the baseline state anxiety item along with the Social Phobia Scale and were randomized to undergo either the speech or interaction task first.

Participants were given details of the first social challenge task and reminded that they had the right to withdraw from the study at any point (no withdrawals occurred). Immediately prior to the social challenge task, participants completed the state anxiety item to assess anticipatory anxiety. Immediately following the task, participants again completed the state anxiety item, retrospectively indicating the anxiety they had experienced during the task. Participants were independently rated on their social performance by the audience of confederates (speech task) or the conversation partner (interaction task) using the SPRS, with ratings not disclosed to participants. This procedure was then repeated with the second social challenge task.

### Statistical analysis plan

The association of social anxiety and sex with observer ratings was examined by conducting separate regression analyses on each SPRS dimension, with predictors of social anxiety, sex (− 1 = males, + 1 = females) and a Social Anxiety X Sex interaction term. Social anxiety was standardized but SPRS ratings were left unstandardized, so that the raw regression coefficient is interpreted as the mean change in rating points (on the 1–5 scale) following a one standard deviation increase in social anxiety. The interaction term was computed by cross-multiplication of sex and standardized social anxiety scores [31].

To determine whether regression coefficients of social anxiety and behavioral ratings differed significantly across the different SPRS dimensions, we tested the equality of these coefficients within a structural equation model. Predictors were the same as for the multiple regression analysis described above, and outcome variables were two SPRS dimensions (specified with correlated errors) whose coefficients were to be compared. We then imposed an equality constraint on the coefficient of social anxiety with each of two performance dimension coefficients. If a likelihood ratio test indicates a significant decrease in fit when an equality constraint is used, this indicates that the two coefficients are not equal [32]. Analyses were conducted in *R* using the *lavaan* [33] package .

## Results

### Data screening

Regression residual plots for SPRS ratings revealed normality and homoscedasticity assumptions were met with no obvious outliers present. A negative skew of speech and interaction task times (due to a ceiling effect from the 3-min time limit) was observed, so *p*-values for analysis of task time data were computed from 10,000 bootstrapped samples.

### Social challenge tasks: anxiety manipulation check

Consistent with the successful induction of anxiety, paired t-tests found significant increases from baseline anxiety for the speech task at pre-task (*t* (92) =5.58, *p* < .001) and during-task (*t* (92) =9.92, *p* < .001) periods, and for the interaction task at pre-task (*t* (92) =5.84, *p* < .001) and during-task periods (*t* (92) =5.69, *p* < .001) (see Table 1 for mean task anxiety scores at each assessment period). To check that anxiety was induced in both male and female participants, t-tests were repeated for each gender separately. For males, significant increases from baseline anxiety were uniformly found at pre-task (*t* (44) =3.61, *p* < .001) and during-task (*t* (44) =5.63, *p* < .001) in the speech task, and pre-task (*t* (44) =2.52, *p* = .015) and during-task (*t* (44) =4.15, *p* < .001) in the interaction task. This pattern of results was replicated for females, with significant increases from baseline anxiety observed at pre-task (*t* (47) =4.49, *p* < .001) and during-task (*t* (47) =8.58, *p* < .001) for the speech task, and pre-task (*t* (47) =5.89, *p* = .015) and during-task (*t* (47) =4.03, *p* < .001) for the interaction task.

**Table 1:** Correlations of social anxiety and sex with anxiety responses

			*Speech Task*		*Interaction Task*	
	SPS	Anx (Base)	Anx (P)	Anx (D)	Anx (P)	Anx (D)
Correlations						
SPS		.47**	.62**	.47**	.58**	.52**
Sex^a^	.24*	.06	.25*	.30*	.24*	.12
*M*	20.0	3.5	4.8	6.0	4.6	5.0
*SD*	11.6	2.0	2.2	2.4	2.2	2.5

*p < .05, **p <. 01

*SPS* Social Phobia Scale, *Anx* Anxiety (Base = baseline, P = pre-task, D = during-task)

^a^Sex coded such that a positive point-biserial correlation indicates greater anxiety for females

Table 1 also reports correlations of social anxiety and gender with self-reported anxiety and shows social anxiety to be consistently moderately associated with increased anxiety response, and additionally that females generally reported greater anxiety compared to males.

Some participants terminated the social challenge tasks before the 3-min limit (speech *M* = 127 *s*, interaction *M* = 177 *s*). As such, we computed the association between social anxiety and task time, as observers’ ratings might conceivably be affected by early task termination. No significant association was observed for either speech (*r* = −.02, *p* = .88) or interaction (*r* = −.19, *p* = .13) tasks.

### Primary analysis

Separate regression analyses were performed on each SPRS dimension for the speech and interaction tasks resulting in 9 regression tests (4 SPRS speech dimensions, 5 SPRS interaction dimensions). To control type I error rate, we used an adjusted alpha criterion of α = .021 based on the Dubey-Armitage Parmar correction [34], which adjusts the conventional level of .05 based on the number of tests conducted (9) and the mean correlation between outcomes (*r* = .59 for SPRS ratings).

### Speech task: social anxiety, sex and SPRS ratings

Table 2 shows the unstandardized (*B*) and standardized (*ß*) coefficients of social anxiety with observer ratings on each SPRS item resulting from the regression analysis of the speech task. These results show that social anxiety was a significant predictor of increased discomfort^2^ (*B* = -0.28, *ß* = -0.42*, p* < .001), but not of gaze, vocal quality or length. There were no significant sex (Table 3) or Social Anxiety X Sex interaction effects (*p* = .10–.96).

**Table 2:** Unstandardized (B) and standardized (ß) regression coefficients of social anxiety with different social performance ratings (negative coefficients indicate higher social anxiety is associated with poorer performance)

		Adequacy of Gaze	Vocal Quality	Length	Low Discomfort	Flow^a^
Speech	*B*	.-18	−.10	−.05	−.28	–
	*95% CI* ^b^	− 0.4, 0.04	−0.3, 0.08	− 0.3, 0.2	−0.44,-0.12	–
	*ß*	−.21	−.15	−.06	−.42	–
	*p*	.115	.267	.674	<.001	–
Interaction	*B*	−.22	−.08	−.06	−.36	−.06
	*95% CI*	−.46, .01	−.30, .14	−.35, .22	−.57, −.16	−.35, .22
	*ß*	−.26	−.09	−.06	−.45	−.06
	*P*	.054	.467	.648	<.001	.658

Performance dimensions: Gaze - adequacy of eye contact; Vocal Quality – warmth, clarity and enthusiasm demonstrated in verbal expression; Length – low level of monosyllabic speech/excessive talking; Discomfort – minimal behavioral anxiety (e.g. fidgeting, trembling); Flow - verbal fluency

^a^Flow item is specific to interaction assessment

^b^*95% CI* = 95% Confidence Interval around B

**Table 3:** Mean (and SD) on each SPRS rating for males and females along with *p*-values for gender from regression analysis

		Adequacy of Gaze	Vocal Quality	Length	Low Discomfort	Flow^a^
Speech						
Males	*M (SD)*	3.51 (.91)	3.45 (.77)	3.50 (1.01)	3.52 (.75)	–
Females	*M (SD)*	3.58 (.78)	3.52 (.67)	3.20 (.75)	3.54 (.60)	–
	*p*	.384	.391	.090	.195	
Interaction						
Males	*M (SD)*	3.83 (.72)	3.38 (.85)	3.59 (1.12)	3.38 (.70)	3.39 (1.06)
Females	*M (SD)*	3.71 (1.00)	3.54 (.83)	3.46 (1.02)	3.51 (.91)	3.36 (1.05)
	*p*	.625	.225	.596	.162	.979

Performance dimensions: Gaze - adequacy of eye contact; Vocal Quality – warmth, clarity and enthusiasm demonstrated in verbal expression; Length – low level of monosyllabic speech/excessive talking; Discomfort – minimal behavioral anxiety (e.g. fidgeting, trembling); Flow - verbal fluency

^a^Flow item is specific to interaction assessment

With respect to the magnitude of the association between social anxiety and SPRS discomfort, as SPRS ratings were left unstandardized, *B* represents the mean change in SPRS discomfort ratings on the 5-point scale for a one *SD* increase in social anxiety. As such, this indicates that a change from − 1 *SD* (low) to + 1 *SD* (high) social anxiety is associated with a 0.56-point increase in discomfort.^2^

### Interaction task: social anxiety, sex and SPRS ratings

For the interaction task, social anxiety was significantly associated with ratings on the discomfort dimension (*B* = -0.36, *ß* = -.45, *p* < .001), but not with other SPRS dimensions (Table 2). No significant sex (Table 3) or interaction effects (*p* = .09–.98) were observed. The unstandardized regression coefficient of *B* = -0.36 for discomfort indicates that a change from − 1 *SD* (low) to + 1 *SD* (high) social anxiety is associated with a 0.72-point increase^2^ in discomfort.

### Comparison of regression coefficients of social anxiety across SPRS dimensions

A likelihood ratio test was used to compare the regression coefficient of social anxiety for SPRS discomfort with regression coefficients for the other SPRS dimensions. For the speech task, the coefficient for SPRS discomfort was significantly greater than all other SPRS dimensions (χ^2^ = 6.56–17.65, all *p*’s < .01). For the interaction task, the coefficient was significantly greater for SPRS discomfort compared to all other SPRS dimensions (χ^2^ = 4.37–5.36, all *p*’s < .05) except SPRS gaze (χ^2^ = 1.31, *p* = .25).^3^

## Discussion

One of the primary findings from this study was that social anxiety was associated with higher observer ratings of behavioral discomfort (e.g., fidgeting, trembling, swallowing) during interaction and speech tasks, but not with other dimensions such as verbal fluency or quality of verbal expression.

Previous research investigating the link between social anxiety and social behavior has produced inconsistent results. It has been suggested that this inconsistency could be partially attributable to differences across studies in the dimension of social behavior assessed, with social anxiety potentially impairing only some behavioral dimensions; although no coherent pattern of which elements of social behavior may be affected has emerged [10]. The current results suggest that, at the non-clinical level at least, social anxiety may magnify the visible signs of anxiety but have little impact on other social behavior dimensions that were assessed here. These results are broadly consistent with Bögels et al. [19] who compared performance ratings for undergraduates low and high in social anxiety. They found that socially anxious participants received significantly more negative ratings on a “showing anxiety symptoms” factor, but not on a “skilled behavior” factor. Similarly, Cartwright-Hatton et al. [14] found that social anxiety scores were significantly associated with observer ratings of nervousness in schoolchildren based on a videotaped two-minute presentation, but not with “overall” impressions of performance (based on three items of ‘cleverness of speech’, friendliness and performance quality). It is difficult to determine from these previous studies if this is indicative of genuine selective effects on visible anxiety signs or simply chance variation, as no statistical comparison across dimensions was made. To our knowledge, the current study is the first to provide a statistical evaluation of these differences. The fact that social anxiety was significantly more strongly associated with behavioral discomfort than the vast majority of all other dimensions suggests that social anxiety in the non-clinical range *is* reliably associated with selective behavioral impairment and that this is confined to manifest and observable signs of discomfort.

It is important to note that not all previous studies are consistent with an effect of social anxiety confined only to overt signs of anxiety. Some studies have found poorer observer ratings of fluency and voice intonation during a speech [15] and vocal clarity and eye contact during a conversation task [16] for patients with SAD compared to controls. However, a tabulated summary of past research findings [10] seems to suggest that where the ‘performance’ aspects of social behavior are also affected, this generally appears to be in clinical samples. The most logical conclusion to draw from this is that high levels of social anxiety within the non-clinical range may primarily exacerbate visible anxiety signs with less impact on other performance aspects, but exhibit broader impairing effects at the clinical level; although it is important to point out this does not appear to have been systematically examined.

The link between social anxiety and discomfort ratings suggests that behavioral signs of anxiety are visible to others during social challenges. If those high in social anxiety engage in safety behaviors to mask their anxiety (e.g., attempting to disguise shaking) as evidence suggests [8], our findings indicate these may have limited effectiveness – at least within the range of social anxiety typically encountered in a non-clinical population. In terms of the magnitude of increased visible anxiety symptoms, those high in social anxiety (one standard deviation above the mean) were rated by observers as approximately half (speech task) to three-quarters (interaction task) of a point higher than those low in social anxiety (one standard deviation below the mean) on the five-point scale used. Determining whether this constitutes a “meaningful” difference is difficult, although the fact that this difference at least approaches a whole-point difference in the scale’s anchor-points (e.g., from “good” to “fair”) is suggestive of a meaningful discrepancy and one that can be demonstrably perceived by others. Overall, these findings clearly show that social anxiety is associated with observable effect on social behavior even in the non-clinical range. Given that a non-clinical sample represents the largest segment of the population, this indicates that social anxiety may have negative effects for a large number of individuals.

The fact that social anxiety failed to be associated with behavioral ratings other than for overt anxiety symptoms is perhaps surprising. Social anxiety scores were strongly correlated with increased anxiety response during social challenges, and the disruptive effect of state anxiety on working memory and the processing of external information including social cues is well supported both theoretically (e.g., via occupation of attentional resources) and empirically [8, 35]. As such, aspects of social behavior expected to involve significant cognitive demands, such as the production of coherent and fluent verbal responses, would seem likely to be impaired. While the lack of association is perhaps unexpected, several possible explanations can be considered. First, the sheer frequency of anxious thoughts in the socially anxious during social challenges could lead to their automatization, so that they fail to consume significant attentional resources to cause cognitive interference [11]. Second, socially anxious individuals are more likely to employ socially facilitative coping strategies, such as overt expressions of enthusiasm or listening to others [9], and this may help compensate for any disruptive effects of anxiety and encourage more favourable impressions of overall social competence. Third, although social anxiety was associated with increased task anxiety for our non-clinical sample, the magnitude of anxiety response needed to produce significant impairment may only be apparent at the clinical level. It should be noted that these explanations for the pattern of effects observed are necessarily speculative and require empirical corroboration.

With respect to sex, while women reported greater anxiety during social challenges, no evidence was found that the link between social anxiety and behavior was more pronounced in females. One recent non-experimental study did report a negative association between social anxiety and self-assessment of social skill in females but not males [36]. The current results suggest that, if such a sex-specific effect on self-assessed social competence is reliable, this does not appear to translate to actual behaviour as rated by others. It is important to treat the lack of any sex-specific influence found here with caution, however, given that interaction effects typically require large sample sizes to detect small or even medium effects. Nevertheless, our findings do suggest that if any such sex-specific effect does exist, this effect is unlikely to be large.

Several limitations of the current study should be noted. First, we used a non-clinical sample, and even if social anxiety does operate on a continuum as is commonly believed [3], results may not generalize to clinical levels of social anxiety. Second, conclusions drawn on the link between social anxiety and social behavior are necessarily limited to the circumscribed set of parameters examined, i.e., molecular indicators of performance during brief social challenges. Findings cannot be automatically assumed to apply to other, perhaps less easily defined or quantifiable facets of performance [6] in more prolonged or situationally different social challenges. Similarly, we used relatively structured tasks with participants given clear instructions on what to do, with evidence suggesting that unstructured situations may cause greater difficulties for socially anxious people [18]. Third, we restricted our study to presentational and interactive scenarios and did not examine situations involving fears of being observed (e.g. eating or drinking) and our results may not generalize to these types of situations. Nevertheless, the tasks employed here are fairly indicative of those commonly encountered outside of the laboratory, with the behavioral indicators believed to represent important features of social competence [27].

Despite these limitations, the current findings have several implications. The fact that social anxiety appears to be most strongly linked to an increase in observable signs of anxiety suggests that techniques directed towards the management of overt anxiety symptoms for those high in social anxiety may be particularly effective for improving impressions of social competence in specific domains where this is likely to be important. Techniques that help the individual recognize their use of anxious behaviors (e.g., throat clearing, fidgeting) and practicing elimination of these in a safe environment [37] may be especially beneficial. Progressive muscle relaxation may also prove useful to reduce muscle rigidity and promote the appearance of a relaxed posture. If successful, these techniques may produce more successful outcomes in situations where reduced signs of anxiety might be considered favorable, such as job interviews or presentations. Such interventions might even contribute to a potential reduction in social anxiety. Specifically, one feature of cognitive models is that socially anxious people tend to excessively focus on and overestimate the occurrence of behavioural, cognitive and somatic responses (e.g. shaking and sweating), and this contributes to a negative mental image of how one appears to others during social encounters [38]. Controlling somatic symptoms which are one source of this attentional focus may promote more positive imagery of one’s projected social self, which has been shown to increase explicit self-esteem [39] and may act as a positive reinforcer of social encounters reducing safety behaviours such as avoidance. It is important to emphasise that we did not investigate such interventions within this study, so these interpretations are entirely speculative. Nevertheless, these processes do represent logical pathways for how techniques directed towards managing visible anxiety signs, that we found to be amplified in those with high social anxiety here, could be potentially beneficial. In addition, the fact that social anxiety was associated with increased observable discomfort in a non-clinical sample also suggests that such management techniques may have potentially widespread benefits to a large sector of the population vulnerable to anxiety in a range of commonly encountered and important social challenges. The apparent selective effect of social anxiety also underlines the need for future studies to include multidimensional assessments of social behavior to fully explicate the nature of the relationship between social anxiety and social behavior.

## Conclusions

In conclusion, the current findings suggest that, the detrimental effects of social anxiety on social behavior within the non-clinical range may be confined to the exacerbation of observable, physical anxiety symptoms with little discernible impact on performance quality. These results underline the necessity of including multiple behavioral dimensions in additional studies and suggest that techniques directed towards the management of outwardly observable anxiety symptoms may be particularly beneficial for socially anxious individuals. Given the importance of everyday “performing” to successful social functioning, research should continue to examine how social anxiety impacts upon social behavior at both the clinical and non-clinical level.

## Abbreviations

ICC: Intraclass Correlation
M: Mean
SAD: Social anxiety disorder
SD: Standard Deviation
SIAS: Social Interaction Anxiety Scale
SPRS: Social Performance Rating Scale
SPS: Social Phobia Scale

## Footnotes

1 We also administered Mattick and Clarke’s companion SIAS scale to provide psychometric data for a separate study. When we substituted the SPS with the SIAS in the current study, there was no impact on the pattern of results.

2 SPRS discomfort is scored such that lower ratings indicate poorer performance (i.e. greater discomfort).

3 We also reran these tests using only one SPRS outcome at a time. This was done as a consistency check to ensure that the results of the hypothesis testing in sections 3.4 and 3.5, which used a regression approach, were the same as those using an SEM approach. As expected, both techniques produced the same results (least squares and maximum likelihood estimators used in regression and SEM respectively produce identical estimates under the usual assumptions of regression).
